# An optimized exosome production strategy for enhanced yield while without sacrificing cargo loading efficiency

**DOI:** 10.1186/s12951-022-01668-3

**Published:** 2022-10-29

**Authors:** Rongxin Zhang, Te Bu, Ruidan Cao, Zhelong Li, Chen Wang, Bing Huang, Mengying Wei, Lijun Yuan, Guodong Yang

**Affiliations:** 1 College of Life Science, Northwest University, Xi’an, 710069 China; 2 The State Laboratory of Cancer Biology, Department of Biochemistry and Molecular Biology, Air Force Medical University, Changlexi Road No.169Th, Xi’an, 710032 China; 3 Department of Ultrasound Diagnostics, Tangdu Hospital, Air Force Medical University, Xi’an, 710038 China; 4 Center of Clinical Aerospace Medicine, Air Force Medical University, Xi’an, 710032 China

**Keywords:** Exosomes, Yield, mRNA cargo, Red cell membrane, Familial hypercholesterolemia

## Abstract

**Background:**

Exosome mediated mRNA delivery is a promising strategy for the treatment of multiple diseases. However, the low yield of exosomes is a bottleneck for clinical translation. In this study, we boosted exosome production via simultaneously reducing the expression of genes inhibiting exosome biogenesis and supplementing the culture medium with red cell membrane components.

**Results:**

Among the candidate genes, knocking down of Rab4 was identified to have the highest efficacy in promoting exosome biogenesis while without any obvious cytotoxicity. Additionally, supplementing red cell membrane particles (RCMPs) in the culture medium further promoted exosome production. Combination of Rab4 knockdown and RCMP supplement increased exosome yield up to 14-fold. As a proof-of-concept study, low-density lipoprotein receptor *(Ldlr)* mRNA was forced expressed in the exosome donor cells and passively encapsulated into the exosomes during biogenesis with this strategy. Though exosome production per cell increased, the booster strategy didn’t alter the loading efficiency of therapeutic *Ldlr* mRNA per exosome. Consistently, the therapeutic exosomes derived by the strategy alleviated liver steatosis and atherosclerosis in *Ldlr*^*−/−*^ mice, similar as the exosomes produced by routine methods.

**Conclusions:**

Together, the proposed exosome booster strategy conquers the low yield bottleneck to some extent and would certainly facilitate the clinical translation of exosomes.

**Supplementary Information:**

The online version contains supplementary material available at 10.1186/s12951-022-01668-3.

## Background

Exosomes (40–150 nm in diameter, with endosome origin) are a kind of lipid bilayer-enclosed nanovesicles secreted into the extracellular space by nearly all types of cells [1]. Exosomes are encapsulated with different biological cargos, such as microRNA, mRNA and protein, transferring the message from the donor cells to the recipient cells [2–4]. Beyond the pathophysiological roles, exosomes are considered as an outstanding vehicle for drug delivery, owing to their physiochemical stability, long blood circulation time, and biocompatibility [1]. As a promising drug delivery carrier, clinical application of exosome faces many challenges, including low yield, purity and loading efficiency [5]. Increasing exosome yield without sacrifice of therapeutic efficacy is badly needed to move to clinical translation.

Exosome biogenesis starts from the plasm membrane invagination to form early endosomes. Early endosomes mature into late endosome, and then the late endosomal membrane invaginates to generate intraluminal vesicles in the lumen of the multivesicular bodies (MVBs). MVBs can either fuse with the plasma membrane and release of intraluminal vesicles as exosome or undergo targeted degradation after fusion with lysosomes [6, 7]. These is also some evidence that supporting membrane exchange between the trans-Golgi network and the endosomal membrane systems [8]. Inward budding of endosome and selection of exosomal cargos are fine-tuned [9, 10], and manipulation of the related pathways is likely to increase exosomes yield.

Several protein classes, such as members of ESCRT and the Rab family, are important for exosome biogenesis [8, 11, 12]. ESCRT is composed of ESCRT-0, EXCRT-I, ESCRT-II, ESCRT-III and associated Vps proteins [9, 13]. It has been revealed that intervention of ESCRT would alter the yield of exosomes [11, 14].

Rab proteins, a largest family of small GTPase proteins, have also been recently considered as a kind of moderators of exosome biogenesis via regulating membrane traffic and vesicle budding [15, 16]. For example, Rab27 plays a significant role in exosomes secretion by docking, tethering and fusing MVBs with the plasma membrane [17]. In addition, Rab31 has been found to promote the production of ILVs during exosomes biogenesis [18]. Besides these exosome promoting proteins, there are also certain proteins that function as exosome biogenesis inhibitors via diverting the exosome for degradation or recycle, such as Rab7 mediated the fusion of MVBs with lysosome during exosome biogenesis [18].

In addition to the endogenous genes involved in exosome biogenesis, the culture condition and microenvironment could also affect exosome yield. For example, ceramide has been found to be a key regulator of exosome secretion [19, 20]. Theoretically, the donor cells with robust secretion of the lipid structured exosomes needs nutrients replenishment [21, 22]. The red blood cells are widely exploited as biological membrane suppliers, because red blood cells are rich in membrane components and easy for membrane particle preparation [23, 24]. Red blood cell membrane particles could be used to replenish the membrane components.

In this study, we boosted exosome production via simultaneously reducing the expression of genes inhibiting exosome biogenesis and supplementing the culture medium with membrane components. As a proof-of-concept study, low-density lipoprotein receptor *(Ldlr)* mRNA was forced expressed and passively encapsulated into the exosomes in the donor cells with Rab4 knockdown and RCMP supplement. Though exosome production per cell increased 14 folds, the booster strategy didn’t alter the loading efficiency of therapeutic *Ldlr* mRNA per exosome. Consistently, the therapeutic exosomes produced by the strategy alleviate liver steatosis and atherosclerosis in Ldlr^−/−^ mice, similar as the exosomes obtained from routine methods. The proposed exosome booster strategy conquers the low yield bottleneck and would possibly facilitate the clinical translation of exosomes.

## Results

### Design of exosome booster by Rab4 knockdown and RCMP supplementation

**Fig. 1:**
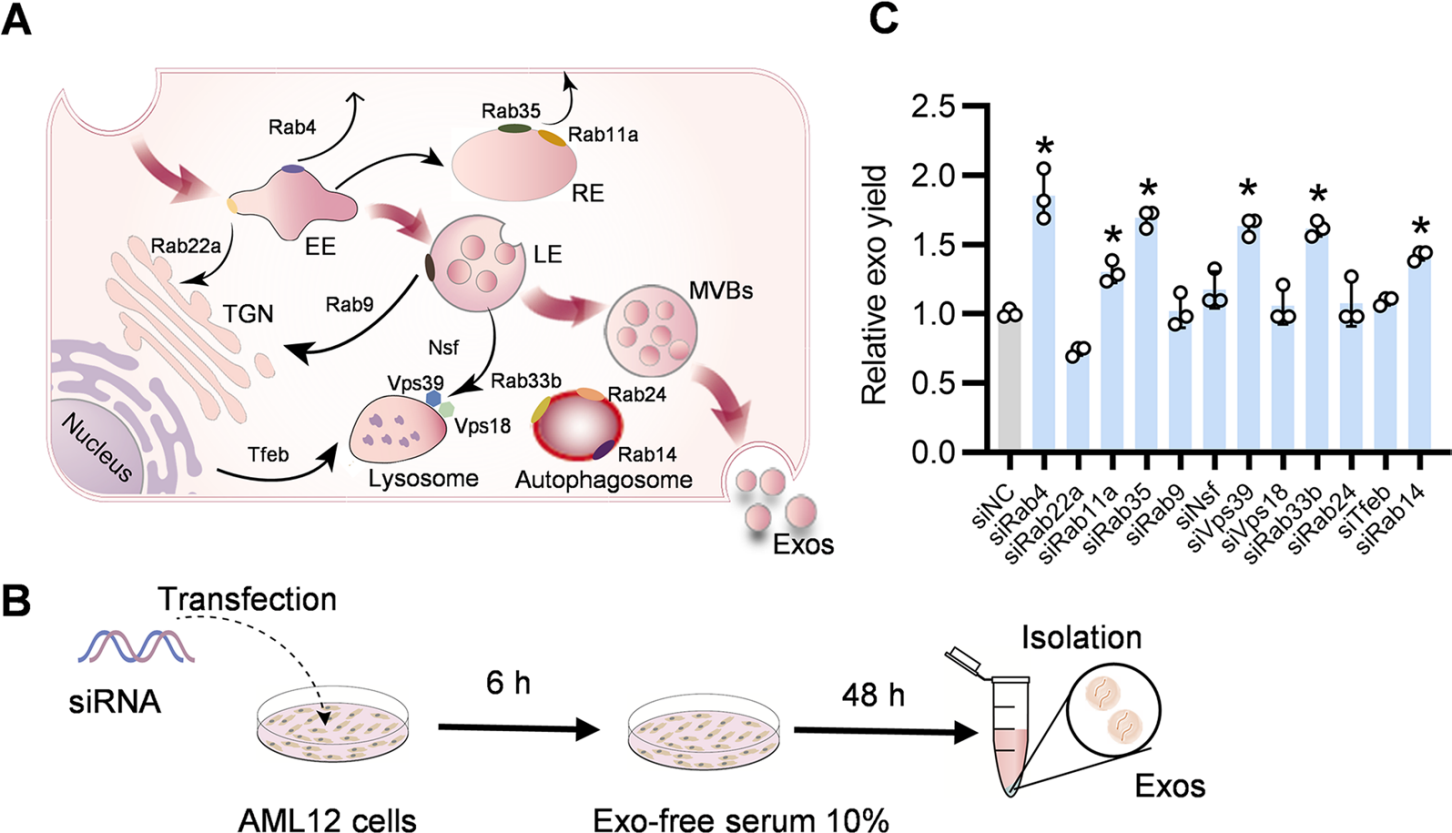
Knockdown of Rab4 effectively increases exosome secretion. **A** Schematic illustration of exosome biogenesis. **B** Schematics illustrating the workflow for screening genes boosting exosome yield. **C** Effects of siRNA on exosome production. AML12 cells were transfected with indicated siRNAs and the derived exosomes were calculated by NTA. All data are expressed as mean $\pm$ SEM of triplicate experiments. *p < 0.05

**Fig. 2:**
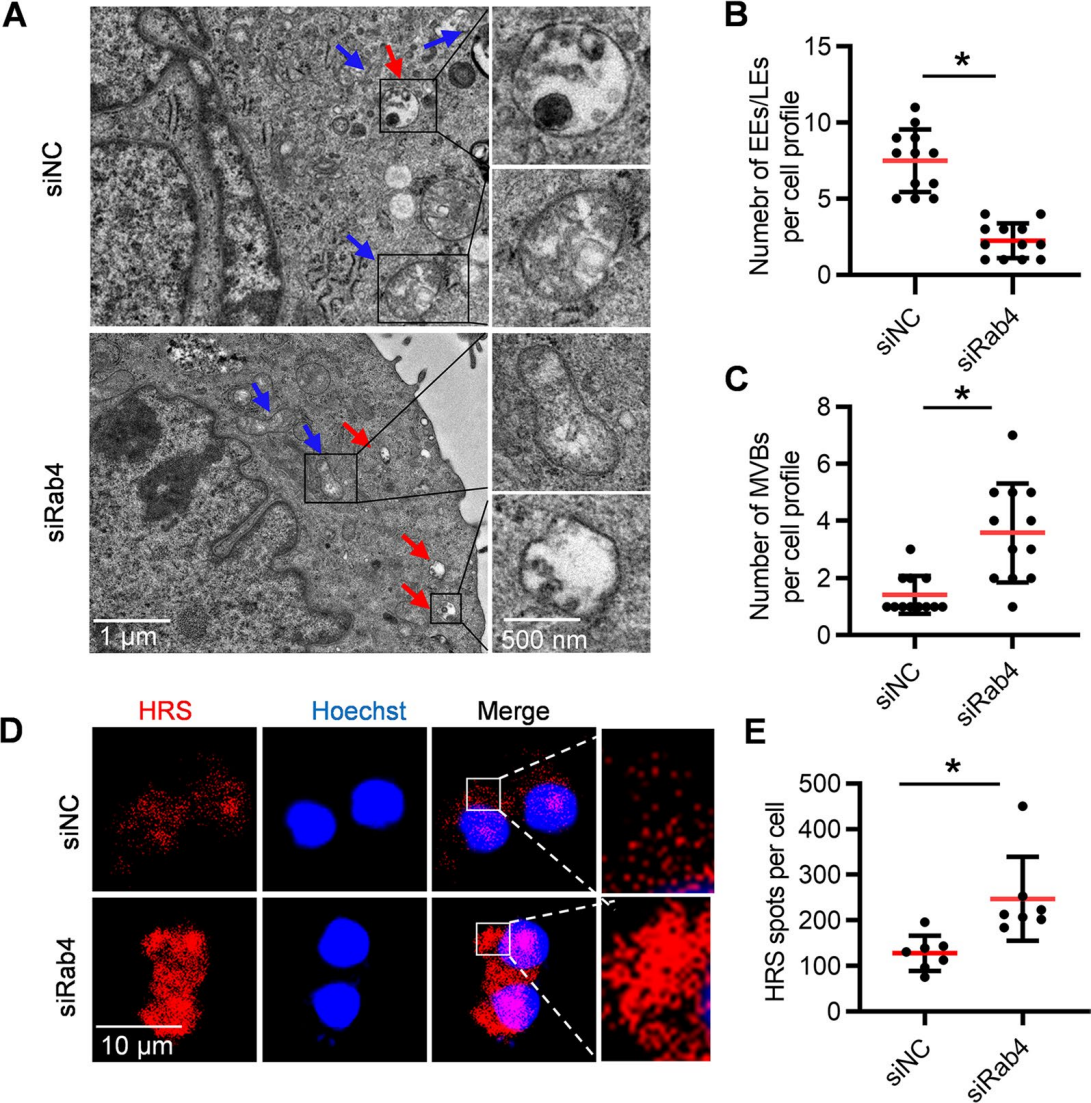
Knockdown of Rab4 increases the number of MVBs. **A** Representative TEM images showing the effects of siRab4 or siNC on exosome biogenesis in AML12 cells. Red arrows indicate MVBs. Blue arrows indicate early endosome (EE)/late endosome (LE). **B** The number of EE and LE per cell profile. Each point represents the number of EE/ LE in each cell profile. **C** The number of MVBs per cell profile. Each point represents the number of MVBs in each cell profile. **D** Confocal microscopy analysis of the HRS in AML12 cells transfected with siRab4 or siNC. **E** Quantification of the number of HRS particles per cell. Each point represents the number of HRS particles from individual cell. All data are expressed as mean $\pm$ SEM. *p < 0.05

The biogenesis and secretion of exosomes are coordinately regulated by many genes (Fig. 1A). To screen genes that inhibit exosome secretion, AML12 cells as the exosome donor cells were transfected with siRNAs against target genes (Fig. 1B). As expected, these siRNAs efficiently reduced expression of corresponding targets at mRNA level (Additional file 1: Fig. S1). Next, we explored whether knockdown of these target genes could increase exosome biogenesis and secretion. Among the candidate genes, knockdown of Rab4 promoted exosome secretion most effectively, as determined nanoparticle tracking analysis (NTA) (Fig. 1C). To explore the potential off-target effects of Rab4 knockdown, we examined Rab31 expression. qPCR analysis showed that Rab4 knockdown didn’t change the expression of Rab31, suggesting that Rab4 knockdown had no obvious off-target effects (Additional file 1: Fig. S2). To further explore the specific mechanism how Rab4 knockdown increases exosome secretion, the morphology and number of endosomes and MVBs in the donor cells were analyzed by TEM. Compared with the control, knockdown of Rab4 decreased the number of early endosomes (EEs) / late endosomes (LEs), while increased the number of MVBs (Fig. 2A–C). Hepatocyte growth factor-regulated tyrosine kinase substrate (HRS), an ESCRT-0 protein, is required for MVBs formation and exosomes secretion [25]. To further verify the positive effect of Rab4 knockdown on exosome biogenesis and secretion, we analyzed clustered localization of HRS fluorescence signals with confocal laser scanning microscope (CLSM). Knockdown of Rab4 resulted in more clustered localization of HRS spots per cell in the cytosol (Fig. 2D, E). These findings suggested that Rab4 knockdown increases exosome biogenesis and secretion by regulating the formation of MVBs.

### RCMP treatment boosts exosome yield

**Fig. 3:**
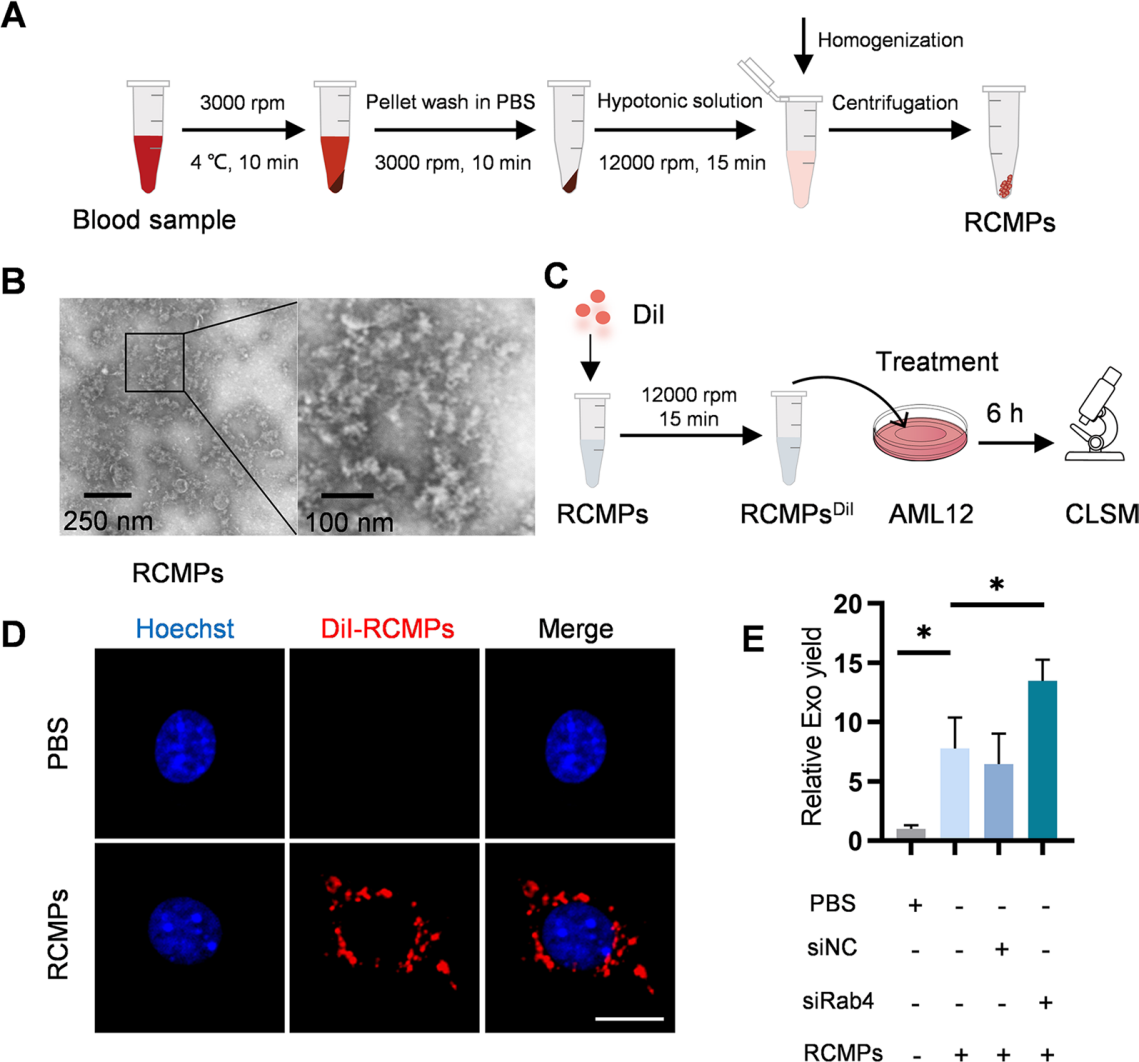
RCMP treatment boosts exosome yield. **A** Schematic illustration of RCMP preparation. **B** Representative TEM images of the prepared RCMP. **C** Schematic diagram of endocytosis of RCMPs into AML12 cells. **D** Fluorescence microscopy images of DiI-labeled RCMP (red) uptaken by AML12 cells. The cell nucleic was stained with Hoechst (blue). Scale bar = 5 μm. **E** RCMP treatment boost exosome yield. AML12 cells were treated with control or RCMPs or RCMPs together with siRNA and the exosomes produced were analyzed by NTA. All data are expressed as mean $\pm$ SEM of triplicate experiments. *p < 0.05

For further optimization, we supplemented the donor cells with red cell membrane particles (RCMPs). RCMPs were prepared from red cells of mice blood (Fig. 3A). RCMPs were nanosized and the morphology appeared irregular in shape under transmission electron microscope (Fig. 3B). To further explored whether RCMPs could be uptake by AML12 cells, AML12 cells were incubated with DiI-labeled RCMPs (Fig. 3C). Fluorescence microscopy analysis revealed that RCMPs could be efficiently endocytosed by AML12 cells (Fig. 3D). Accordingly, flow cytometry analysis further confirmed that most of the AML12 cells treated with DiI-labeled RCMPs had high DiI fluorescence signal (Additional file 1: Fig. S3). As expected, RCMPs supplementation additionally increases exosome secretion, which could be further augmented by Rab4 knockdown (Fig. 3E). Moreover, compared with the controls, Rab4 knockdown and RCMP supplementation didn’t alter the size and morphology of the exosomes (Additional file 1: Fig. S4A, B). Western blot of the exosome inclusive markers CD63 and TSG101 and exclusive marker GM130 further confirmed the exosome identities of the derived extracellular vesicles (Additional file 1: Fig. S4C).

### The booster strategy doesn’t alter the in vivo distribution profile of exosomes in *Ldlr*^*−/−*^ mice

**Fig. 4:**
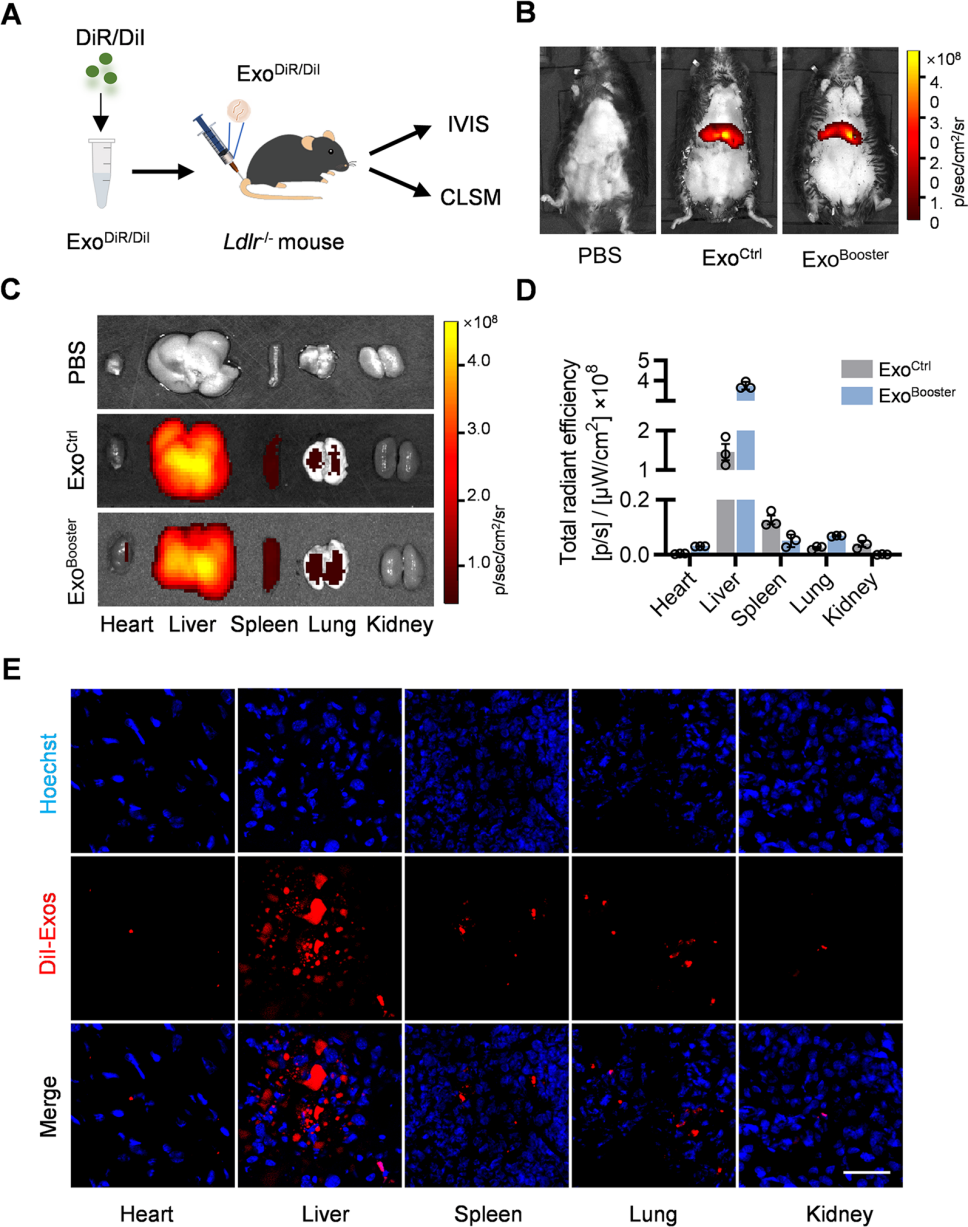
Biodistribution of Exo^Booster^ in vivo. **A** Schematic illustration of the experimental procedure. **B** Representative IVIS images showing the distribution of the exosomes in vivo. Mice were injected with PBS or 100 μl DiR-labeled exosomes via tail vein. IVIS imaging was performed 4 h after injection. **C** Representative IVIS images of the DiR-labeled exosomes in different organs, including the heart, liver spleen, lung and spleen. **D** Quantification of the DiR signal intensity in Fig. 3C. n = 3. **E** Representative fluorescence microscopic images showing the distribution of exosomes in the tissue sections. Mice were injected with PBS or 100 μl DiI-labeled exosomes and different organs were harvested for tissue sectioning. The nuclei were counterstained with Hoechst. Scale bar = 50 μm

To explore the distribution of exosomes in vivo, DiI/DiR-labeled Exo^Ctrl^ (Exosomes from siNC + PBS treated cells) and Exo^Booster^ (Exosomes from siRab4 + RCMPs treated cells) were injected via tail vein, and traced by in vivo imaging system (IVIS) or confocal microscopy (Fig. 4A). Similar distribution profiles between Exo^Ctrl^ and Exo^Booster^ were found, with the DiR or DiI signal enriched mainly in liver and spleen (Fig. 4B–E). Briefly, mice were injected with DiR-labeled exosomes and the in vivo fluorescence signal was detected by the in vivo imaging system. DiR signal was mainly localized in the liver (Fig. 4B), suggesting the liver dominant localization of the exosomes. Then the main organs were separated and imaged, further confirmed the distribution profile among organs (Fig. 4C, D). To further explore the exosome localization in different tissues, mice were injected with DiI-labeled exosomes and the distribution of exosomes were examined by confocal microscopy. Robust DiI signal could be seen in the liver sections (Fig. 4E, Additional file 1: Fig. S5). Together, all the data indicated that exosomes could be delivered into liver.

### No alteration of the cargo loading efficiency by the booster strategy

**Fig. 5:**
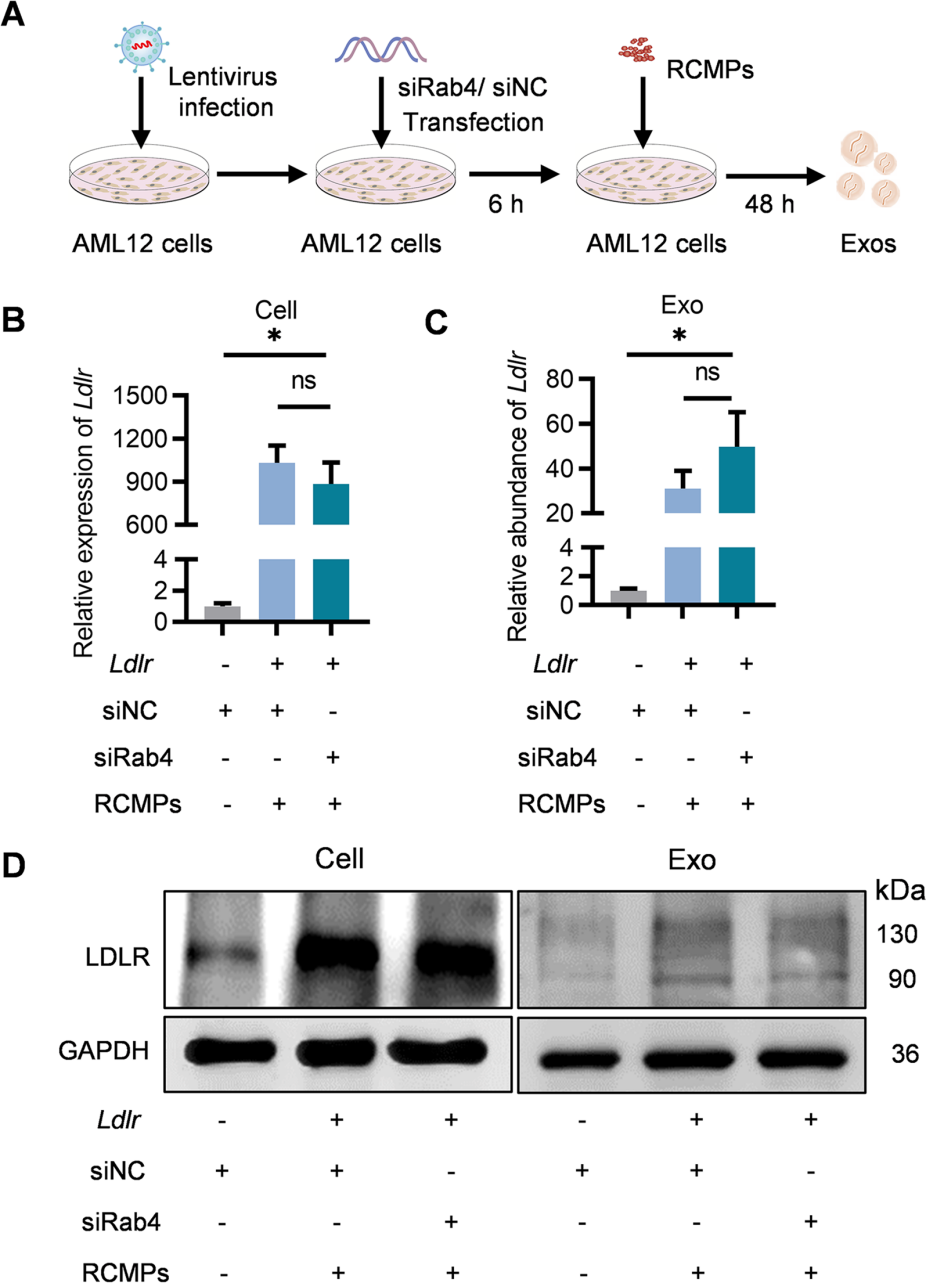
Booster strategy has no obvious effects on cargo loading efficiency. **A** Schematic illustration of the experimental procedure how *Ldlr*@Exo^Booster^ was prepared. **B** qPCR analysis of *Ldlr* mRNA expression in AML12 donor cells as indicated. **C** qPCR analysis of *Ldlr* mRNA in the isolated exosome as indicated. *Gapdh* as an internal reference gene. Data are expressed as mean $\pm$ SEM of three independent experiments. *p < 0.05 by one-way ANOVA. **D** Western blot analysis of LDLR protein level in AML12 cells and the derived exosomes. GAPDH served an internal control. Representative data from three independent experiments

Efficient cargo loading is prerequisite for exosome-based therapy. We next explored whether the booster strategy would sacrifice the loading efficiency. AML12 cells were infected with *Ldlr* overexpressing lentivirus were additionally treated with control or siRab4/RCMPs, with the derived exosomes denoted as *Ldlr*@Exo^Ctrl^ and *Ldlr*@Exo^Booster^ respectively (Fig. 5A). Of note, additional treatment of siRab4 and RCMPs didn’t alter the *Ldlr* abundance in the donor cells, nor the abundance per exosome (Fig. 5B, C). These data indicated the booster strategy didn’t affect the loading efficiency. Besides the mRNA level, there was also slight LDLR protein appeared in the derived exosomes (Fig. 5D).

**Fig. 6:**
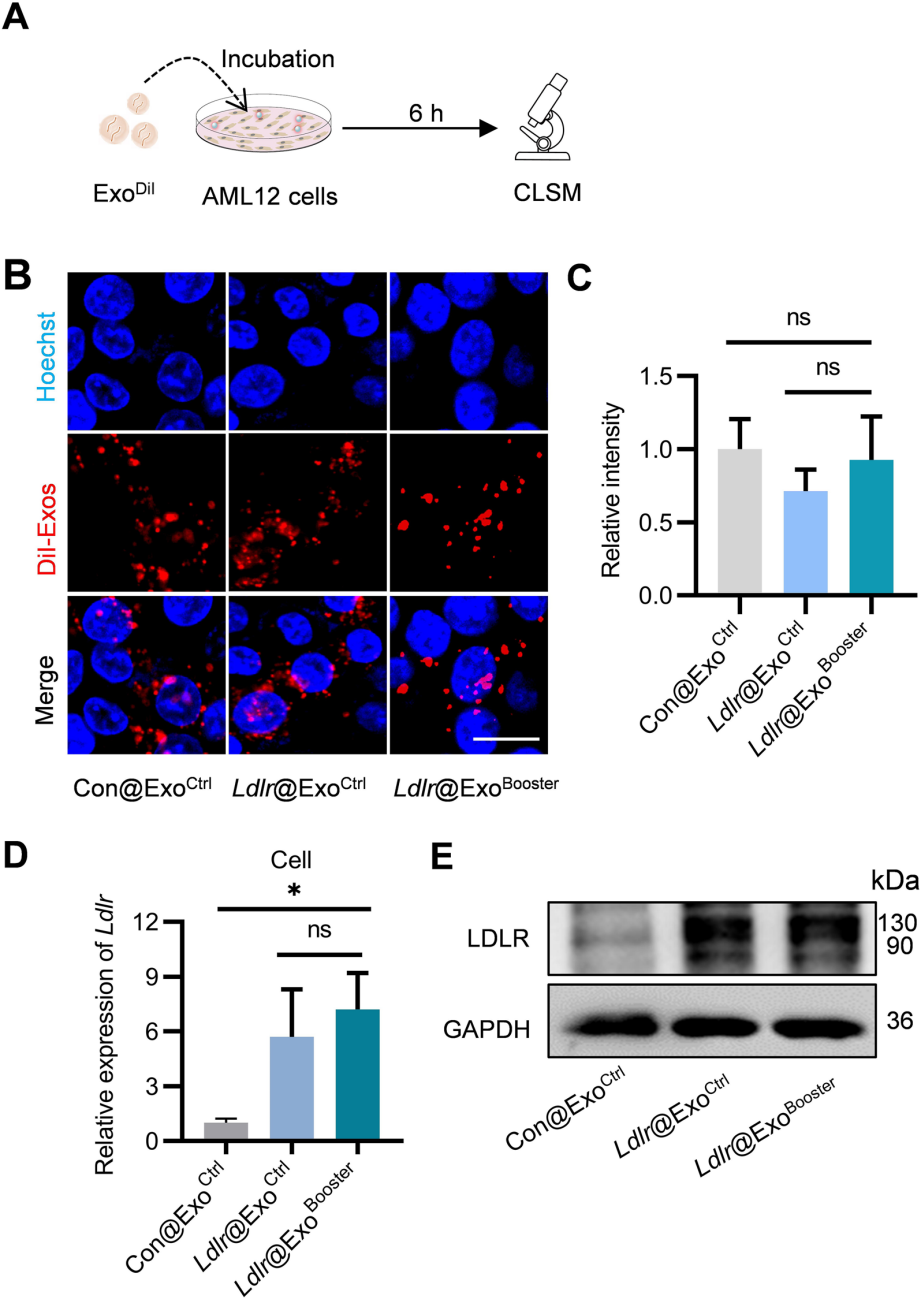
*Ldlr*@Exo^Booster^ efficiently delivers therapeutic *Ldlr* mRNA into target cells. **A** Schematic diagram of the experiment. **B** Fluorescence images demonstrating the endocytosis of exosomes by the recipient cells. The distribution of DiI-labeled exosomes in AML12 cells were imaged by confocal microscopy, with the nuclei counterstained by Hoechst. Scale bar = 10 μm. **C** Fluorescence intensity of DiI signal corresponding to panel B. **D** qPCR analysis of *Ldlr* mRNA level in recipient cells. Data are expressed as mean $\pm$ SEM of three independent experiments. *p < 0.05 by one-way ANOVA. **E** Western blot analysis of LDLR expression at protein level in AML12 cells. Images are representative of three independent experiments

To examine whether *Ldlr*@Exo^Booster^ could effectively deliver therapeutic *Ldlr* mRNA to target cells and allow efficient translation, AML12 cells were treated with Con@Exo^Ctrl^ (exosomes derived from cells infected with control lentivirus and treated with control siRNA), *Ldlr*@Exo^Ctrl^, and *Ldlr*@Exo^Booster^ (Fig. 6A). Fluorescence microscopy revealed that exosomes were effectively endocytosed by AML12 cells for all the tested exosomes, suggesting that engineering of *Ldlr*@Exo^Booster^ didn’t change the endocytosis efficiency (Fig. 6B, C). qPCR analysis and Western blot analysis revealed that *Ldlr*@Exo^Booster^ treatment significantly increased the *Ldlr* mRNA and protein expression in the recipient cells, similar as the *Ldlr*@Exo^Ctrl^ (Fig. 6D, E).

### *Ldlr*@Exo^Booster^ treatment ameliorates liver damage and atherosclerosis in *Ldlr*^*−/−*^ mice

**Fig. 7:**
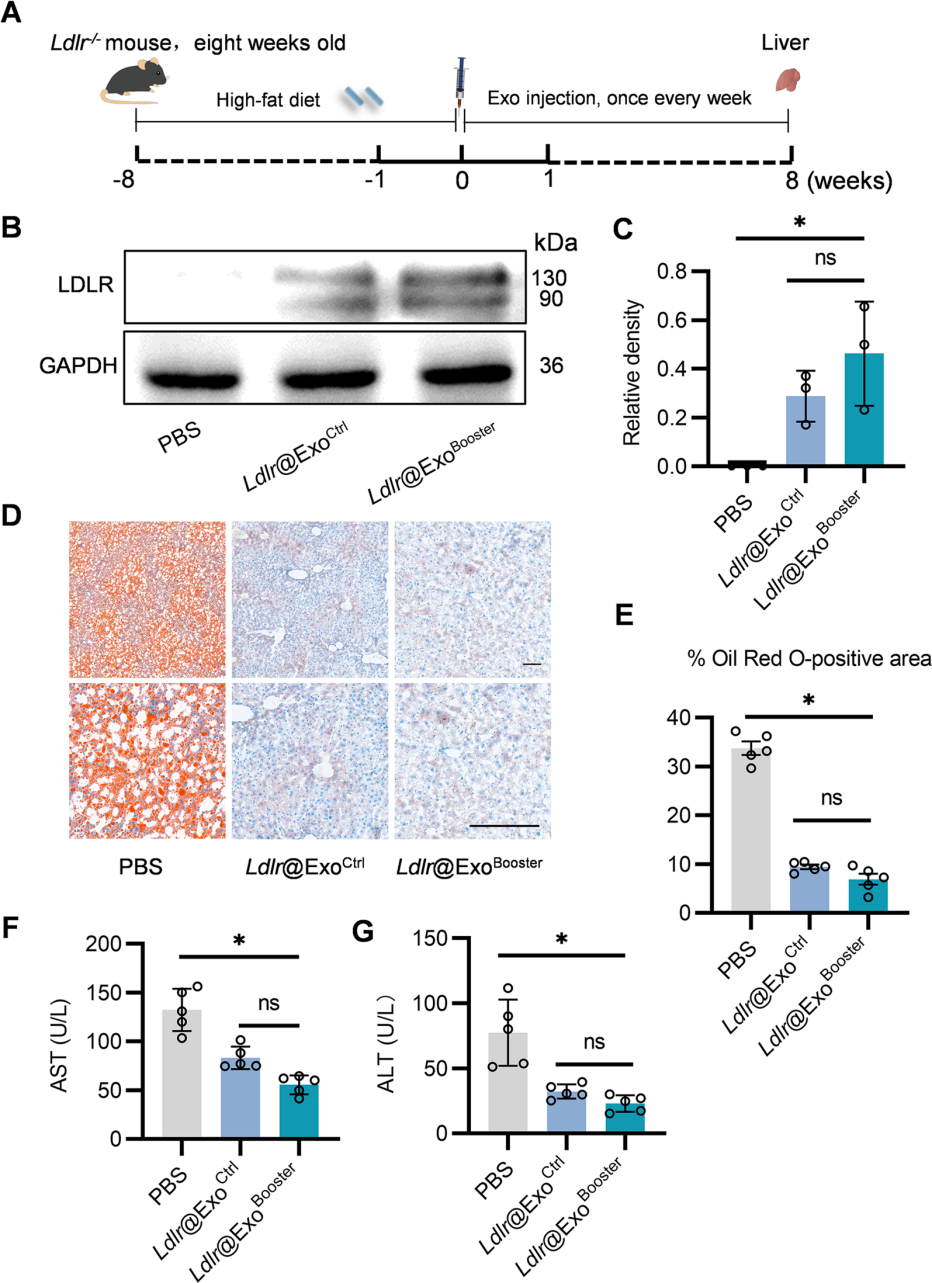
*Ldlr*@Exo^Booster^ alleviates liver damage in *Ldlr*^*−/−*^ mice. **A** Schematic illustration of the experimental procedure. *Ldlr*^*−/−*^ mice were fed with high fat diet for 8 weeks, followed by PBS or exosome treatment once a week for 8 weeks. At the end of the experiments, mice were sacrificed and the liver tissues were harvested for systemic analysis. **B** Western blot analysis of LDLR protein expression in livers from mice treated as indicated. Data shown are representative of three independent experiments.** C** Quantification of western blot bands by densitometry. *P < 0.05 by one-way ANOVA. **D** Representative images of Oil Red O staining in liver slices from mice with indicated treatments. Scale bars = 50 μm. **E** Percentage of Oil Red O positive area in liver sections. Data are expressed as mean $\pm$ SEM. **F**, **G** Serum AST (**F**) and ALT (**G**) levels in the mice treated as indicated. *p < 0.05 by one-way ANOVA. n = 5. AST, aspartate aminotransferase; ALT, Alanine Aminotransferase

In the following experiments, we explored the therapeutic effects of *Ldlr*@Exo^Booster^ *in Ldlr*^*−/−*^ mice. *Ldlr*^*−/−*^ mice were fed with high-fat diet for 8 weeks, and then tail vein injected with *Ldlr*@Exo^Booster^ or the controls at 4 μg/g once a week for 8 weeks (Fig. 7A). Western blot results showed that *Ldlr*@Exo^Ctrl^ and *Ldlr*@Exo^Booster^ treatment robustly restored LDLR protein to a similar level in liver (Fig. 7B, C), while slightly rescued LDLR protein expression in lung and kidney (Additional file 1: Fig. S6A–D). No obvious LDLR protein expression was found in the heart (Additional file 1: Fig. S6E, F). With LDLR restoration, *Ldlr*@Exo^Ctrl^ and *Ldlr*@Exo^Booster^ significantly reduced lipid deposition in the liver, with nearly the same therapeutic effects, as revealed by Oil Red O staining (Fig. 7D, E). Accordingly, blood AST and ALT activity were also reduced to similar levels by *Ldlr*@Exo^Ctrl^ and *Ldlr*@Exo^Booster^ (Fig. 7F, G). Moreover, blood biochemical test results showed that *Ldlr*@Exo^Ctrl^ or *Ldlr*@Exo^Booster^ treatment significantly reduced total triglycerides, total cholesterol and LDL cholesterol (Additional file 1: Fig. S7A–D). These data suggested that *Ldlr*@Exo^Ctrl^ and *Ldlr*@Exo^Booster^ treatment had similar beneficial effects on liver metabolism on a per exosome basis.

### *Ldlr*@Exo^Booster^ treatment ameliorates atherosclerosis in *Ldlr*^*−/−*^ mice

**Fig. 8:**
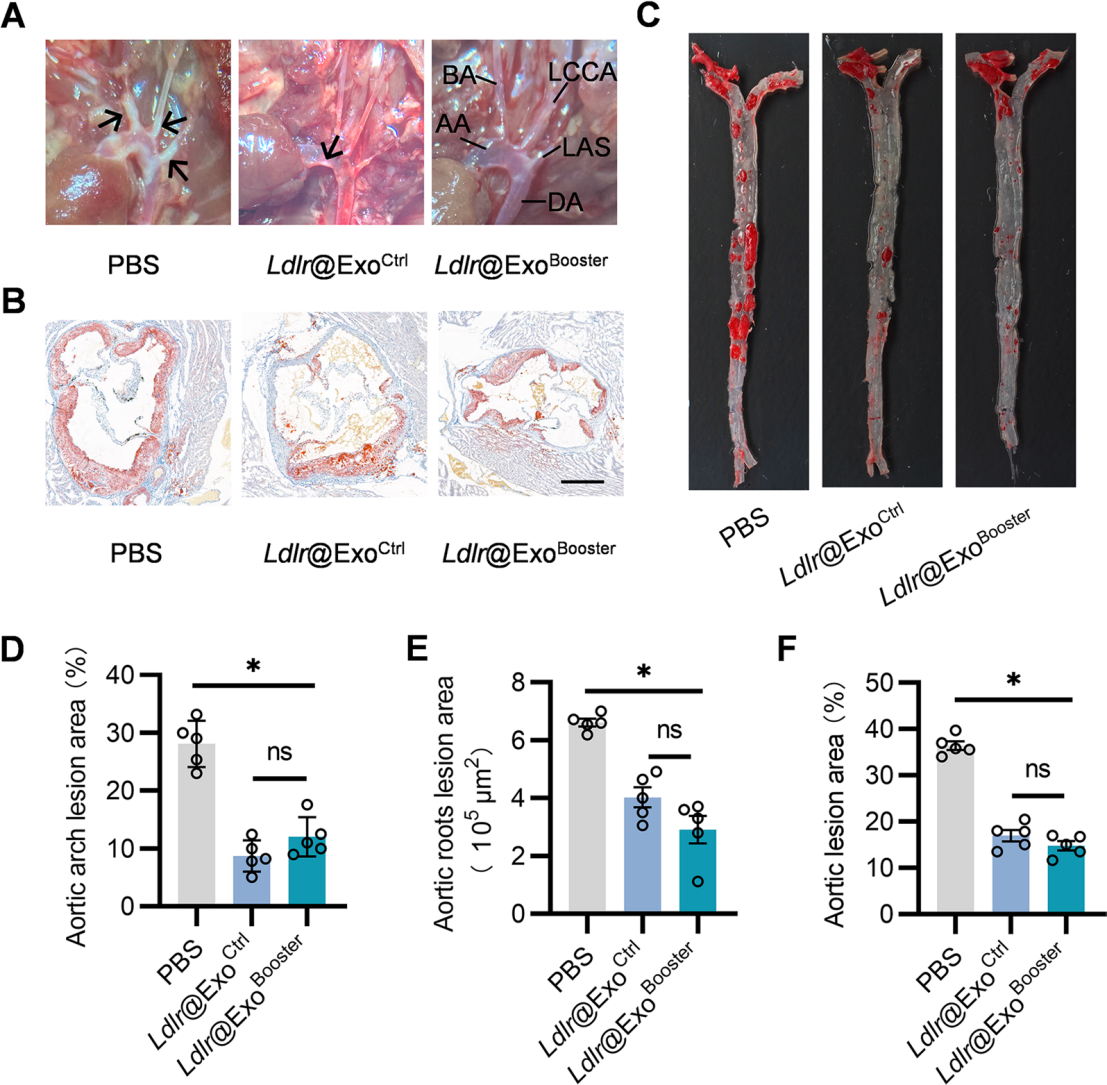
*Ldlr*@Exo^Booster^ treatment ameliorates atherosclerosis in *Ldlr*^*−/−*^ mice. **A** Representative aortic arch view of atherosclerotic lesions in *Ldlr*^*−/−*^ mice treated with PBS or indicated exosomes. AA, ascending aorta; BA, brachiocephalic artery; DA, descending aorta; LCCA, left common carotid artery; LSA, left subclavian artery. **B** Representative images of Oil Red O staining of the aortic roots cross section of *Ldlr*^*−/−*^ mice with indicated treatments. Scale bar = 400 μm. **C** Representative images of en face Oil Red O staining of the plaques in aortas from *Ldlr*^*−/−*^ mice with indicated treatments. **D** Percentage of atherosclerotic area in aortic arch region of mice with indicated treatments corresponding to **A**. **E** Statistical data of the Oil Red O positive plaque area corresponding to **B**. **F** Percentage of the atherosclerotic region corresponding to **C**. All data are showed as mean $\pm$ SEM. *p < 0.05 by one-way ANOVA, n = 5. Schematic illustration of the study: In donor cells, the therapeutic exosomes with *Ldlr* mRNA encapsulated were boosted by Rab4 knockdown and red cell membrane particle supplementation, without any sacrifice on loading efficiency. This strategy could restore *Ldlr* expression and reverse phenotype in *Ldlr *^*−/−*^ mice

In accordance with the lipid metabolism remodeling, *Ldlr*@Exo^Ctrl^ and *Ldlr*@Exo^Booster^ treatment also reduced the number and size of atherosclerotic plaques (Fig. 8A, D). In addition, Oil Red O staining of aortic roots and aortas revealed that the lipid core in the plaque were also less and smaller after *Ldlr*@Exo^Ctrl^ and *Ldlr*@Exo^Booster^ treatment (Fig. 8B, C, E, F)

There was no signification change in body weight after *Ldlr*@Exo^Ctrl^ and *Ldlr*@Exo^Booster^ treatment compared with the control group (Additional file 1: Fig. S8A). And H&E staining showed that *Ldlr*@Exo^Ctrl^ and *Ldlr*@Exo^Booster^ treatment had no obvious effects on the histology of heart, liver spleen, lung and kidney (Additional file 1: Fig. S8B). It is important to note that siRab4 sequence was also encapsulated into the exosomes (Additional file 1: Fig. S9). No obvious toxic effects observed in the *Ldlr*@Exo^Booster^ treatment group could be explained in the following two reasons. Different from mRNAs, siRNA functions in a dose-dependent manner, and thus the abundance of the siRNA might not achieve the threshold. Alternatively, the detection method for side effects is not sensitive enough. Anyway, developing strategies to trap siRab4 in donor cells and restricting it from sorting into exosomes are needed for minimized potential side effects. Together, all of these data confirmed the biocompatibility of the engineered exosomes.

## Discussion

In this study, we have successfully developed an exosome booster strategy, which increase yield of exosomes 14-fold, simply by knocking down Rab4 and supplementing RCMPs in the donor cells. Moreover, the booster strategy neither change the loading efficiency, nor compromise the therapeutic efficacy.

Exosomes hold great promise in the field of targeted drug delivery [7, 26]. At present, the bottleneck of exosome clinical translation is the low yield. Boosting exosome yield is intensively explored recently. Exosome biogenesis is a complex vesicle trafficking process in the cells [1]. Rab27a and Rab27b have been found to have a role in the docking of MVBs with the plasma membrane, which regulates exosomes secretion [17]. Rab31 can promote exosomes secretion by driving the formation of ILVs and inhibiting the degradation of MVBs during the exosome biogenesis process [18]. Enhancing the expression of these genes would probably increase exosome yield. In fact, it has been shown that simultaneous overexpression of STEAP3, Syndecan-4 and a fragment of L-aspartate oxidase significantly increase the secretion of exosomes in a designer exosome strategy [21]. Different from the forced expression of the exosome boosting genes, we here increased the yield of exosomes by reducing the expression of genes inhibit exosome biogenesis and secretion. As we know, Rab4 is a member of the Ras superfamily of small GTPases [27], which plays an important role in regulating membrane trafficking [28]. Briefly, Rab4 protein is involved in the sorting and recycling of early endosomes, tipping the balance from late endosome maturation to quick recycling [29]. Early endosome either fuses with plasma membrane for recycle or matures into late endosome. Inward budding of the late endosome produces ILVs, and then the late endosome matures into the MVB. When MVBs fused with the cellular plasma membrane, the vesicles in the MVB are released as exosomes [1]. When blocking Rab4, the early endosome favors the exosome biogenesis pathway. Among the candidate genes, we revealed that knockdown of Rab4 has the most striking role in promoting exosome biogenesis and secretion. The results could be explained by the fact that Rab4-mediated rapid recycling pathway directly divert the early endosome and to the plasma membrane [30].

In addition, exosome biogenesis also affected by culture conditions [31]. In this study, we supplemented the cells with RCMPs, which further augment exosome production. The results could be explained by the fact that sufficient nutrients are required for boosting exosome production potency. Exosomal membrane has similar lipid-components as the red cell membrane [32]. In order to meet the demand of exosome secretion, cells need a large amount of membrane lipids. Supplementation of RCMPs directly meets the need. In addition, the RCMPs are endocytosed by the cells, which the starting step of exosome biogenesis [33]. This strategy opens a window for boosting exosome yield simply via optimizing the gene expression related to exosome biogenesis and the culture conditions. The proposed strategy is also facile, cost-effective and scalable. We foresee this exosome boosting strategy could open a new window for development of exosome-base therapeutic. It is worthy to investigate whether our strategy is compatible with the previous designer exosome strategy [21], and it is also interesting to explore whether combination of the two strategies further augment the exosome production. Screening for more exosome biogenesis related genes, with maximized effects on exosome yield and minimal effects on cell viability, is still needed. In addition, it should also be concerned whether this strategy is cell type specific or universal.

As a drug carrier, exosomes deliver the encapsulated cargos to adjacent or distant cells, regulating gene expression and modify of phenotypes in the recipient cells [21, 34]. Efficient loading of the therapeutic cargos is prerequisite for therapy. In other words, the boosting strategy should not compromise the loading efficiency. In our study, the excessive *Ldlr* mRNA were passively loaded into exosomes in donor cells, without sacrificing the loading efficiency, which can be explained by the following possibilities: (1) With the exosome yield further increased, the therapeutic mRNA abundance should be further enhanced as feedback; and (2) Alternatively, the cargos in exosomes are encapsulated during endosome sorting process [35], and the therapeutic mRNA could be selectively enriched when excessively expressed.

As to the intracellular delivery of the mRNA encapsulated in the exosomes, the endocytosis pathway might be the dominant manner [36]. Briefly, the mRNAs encapsulated in exosomes are delivered into recipient cells when the exosomes are endocytosed. Following uptake, the exosomes are sorted into endosomes, where exosomal membrane fuses with the endosomal membrane, releasing the mRNA into the cytoplasm for translation. The mRNAs could be also delivered into recipient cells when the exosomes fuse with the plasma membrane.

In this study, RCMPs supplementation (culture condition), Rab4 blocking, mRNA packaging, and exosome isolation are independent procedures and each step is necessary for any exosome-based therapeutic strategy. Optimization of each step is critical for overall efficiency and final clinical translation. Our strategy simultaneously targeting independent steps has achieved a rational efficiency and further modifications should be also explored to integrate with the current strategy. Simultaneous increasing the exosome yield, cargo selective encapsulation, and homogeneity are especially needed for clinical translation.

## Conclusions

In summary, we here boosted exosome production without any sacrifice of therapeutic efficacy via simultaneously reducing the expression of genes inhibiting exosome biogenesis and supplementing the culture medium with membrane components. Though exosome production per cell increased, the booster strategy didn’t alter the loading efficiency of therapeutic mRNA per exosome when the mRNA was forced expressed in the donor cells. Together, the proposed exosome booster strategy conquers the low yield bottleneck in the field to some extent and would highly possible to facilitate the clinical translation of exosomes.

## Materials and methods

### Cell culture

Alpha mouse liver 12 (AML12) cells and HEK293T cells were cultured in high glucose DMEM medium (Logan, Utah, U.S.A.) containing 10% exosome-free FBS (fetal bovine serum), 1% L-glutamine and 1% penicillin–streptomycin (Logan, Utah, U.S.A.) in a humidified atmosphere at 37 °C with 5% CO_2_. The exosome depleted FBS was obtained by removing exosome with ultracentrifugation at 120,000*g* for 3 h at 4 °C (Beckman Coulter X-90 centrifuge, SW41 Ti rotor).

### siRNA transfection

AML12 cells were transfected with scramble siRNA and siRNA target genes of interest by using HiGene transfection reagent (C1506, Applygen Technology Inc.) according to manufacturer’s instruction. The designed sequences of si-NC, si-Rab4, si-Rab22a, si-Rab11a si-Rab35, si-Rab9, si-Nsf, si-Vps39, si-Vps18, si-Rab33b, si-Rab24, si-Tfeb and si-Rab14 (GenePharma) were listed in Additional file 1: Table S1.

### Plasmid construction, lentivirus package and infection

Ldlr coding regions were cloned into pWPI vector replacing the IRES-EGFP as described previously [37]. The *Ldlr* expressing vector was transfected into HEK293T cells together with psPAX2 and pMD2G at the molar ratio of 4:3:1 with HighGene transfection reagent (ABclonal). Lentivirus particles were harvested from the supernatant filtered through 0.45 μm filters 72 h after transfection and stored at − 80 °C. For infection, AML12 cells cultured in plates were incubated with the lentivirus at the MOI of 200 in the presence of polybrene (8 μg/ml).

### Red cell membrane particles preparation

Whole blood samples were collected from the orbit of male mice (C57BL/6) aged 6–8 weeks with the addition of 1.5 mg of EDTA for anticoagulation purpose. Blood samples were centrifuged at 3000 rpm/min for 10 min at 4 °C to remove the plasma and collected RBCs were washed with pre-cooled 1× PBS for five times. Then, 0.1× PBS (PBS: deionized H_2_O = 1:9) was added and placed at 4 °C for 2 h for hemolysis. The released hemoglobin was removed by centrifugation at 12,000 rpm for 15 min, and the pellet was collected and washed for 5 times until the pellet turned light pink color. The red cell membrane pellets were re-suspended with a hydration solution consisted of 1.8 ml of 1× PBS and 200 μl glycerol, followed by homogenization 10 min at 10,000 rpm with a miniature high-speed dispersing homogenizer (F6/10, jingxin technology, shanghai) under ice water bath.

### Labeling of RCMPs and tracking of cellular uptake

The RCMPs were incubated with DiI at 37 °C for 20 min in dark, and transfer to 4 °C for 10 min, then centrifuged at 4 °C for 12,000*g* for 15 min to remove the unbound dye. The labeled RCMPs were resuspend in PBS prior to use. AML12 cells were incubated with DiI -labeled RCMPs for 6 h. Then, the medium was removed and AML12 cells were washed twice with PBS. Cellular internalization of DiI-labeled RCMPs were analyzed by laser scanning confocal microscope or flow cytometer (Beckman CytoFLEX).

### Exosome isolation and characterization

AML12 cells with transfection/infection were cultured in DMEM medium. For supplementation of RCMPs, cells were added with RCMPs (80 μg/ml) and incubation for 6 h before switch to exosome-free medium for additional culture of 48 h. Cells were discarded by centrifugation at 500*g* for 10 min and the residual cellular debris were removed by centrifugation at 5000*g* for 20 min. The collected supernatants were filtered through 0.22 μm filters, and then were ultracentrifuged at 100,000*g* for 2 h (Beckman Coulter X-90 centrifuge, SW41 Ti rotor). The isolated exosomes were resuspended in 1× PBS and stored at − 80 °C till use. Size distribution and concentration of exosomes were analyzed by ZetaView^®^ instrument (Particle Metrix, USA). The samples were loaded into the sample chamber at ambient temperature. Then, the concentration was calculated according to the dilution fold.

For transmission electron microscopy analysis of the exosome morphology, isolated exosomes were allowed to be fixed for 6 h in 2.5% glutaraldehyde in phosphate buffer at 4 °C. Then the samples were dried on a copper grid 5 min, followed by immediate observation at JEM-2000EX electron microscopic analysis (HITACHI, HT7800/HT7700).

### Electron microscopy and MVB quantification

AML12 cells transfected with si-NC and si-Rab4 were washed with PBS and fixed with 4% paraformaldehyde at room temperature. Then, the cells were added onto grid, stained (2% uranyl acetate) and imaged by electron microscopy (HT7800, Hitachi). MVBs numbers per profile were calculated.

### Immunofluorescence

For immunofluorescence assay, AML12 cells with indicated treatments were cultured in confocal dish (35 mm) and incubated for 48 h. Then, the culture medium was discarded and the cells were washed with PBS, followed by fix with 4% paraformaldehyde for 20 min. Then, cells were stained with primary antibody (anti-HRS, sc-271455) overnight, followed by secondary antibody [Goat anti-mice 633, Invitrogen, A-21050)] at room temperature for 1 h in the dark. Finally, the nuclei were stained with Hoechst (1:1000). Images were processed using laser scanning confocal microscope. HRS spots per cell were calculated using Image J.

### In vitro and in vivo tracking of exosomes

Exosomes were labelled with DiR/DiI by direct incubation with the dye (1 μM in final concentration, Invitrogen, China) at 37 °C for 10 min, and then the free dye was removed by centrifugation at 12,000 rpm for 10 min, and the precipitate was re-suspended with PBS.

For in vitro experiment, AML12 cells were seeded into confocal dish and incubated with DiI-labeled exosomes (final concentration of 40 μg/ml) for 6 h. Cells were then washed with PBS and fixed in 4% paraformaldehyde for 10 min at room temperature. The nuclei were stained with Hoechst (C1022, Beyotime, China) for 10 min at room temperature, followed by PBS three times. Cellular uptake of exosomes in vitro was observed by confocal microscopy (Nikon, Tokyo, Japan). The whole experiment was kept in dark.

For in vivo tracing, mice were intravenously injected with freshly prepared DiR or DiI-labeled exosomes samples. After 6 h, the DiR fluorescence signal of the whole mouse and major organs (heart, liver spleen, lung and kidney) were imaged by the in vivo imaging system (IVIS, PerkinElmer, Thermo Fisher, USA), and the DiI fluorescence signal was imaged by confocal microscopy on the tissue sections.

### Western blotting

Protein lysis from cells, exosomes and tissues were prepared and the protein concentration was determined by Pierce BCA Protein Assay Kit (Thermo Fisher Scientific, Waltham, USA.). Protein Samples were separated by 10% or 12% SDS-PAGE gels and transferred to nitrocellulose filter membranes. The nitrocellulose filter membranes were blocked with 3% skim milk in tris buffered saline (TBS) containing 0.1% Tween-20 (TBST) at 4 °C overnight, and then incubated with primary antibodies followed by a horseradish peroxidase-conjugated secondary antibodies (washed with TBST three times before each operation, 5 min each time). Primary antibodies used were anti-LDLR (10785-1-AP, Proteintech), anti-GM130 (sc71166, Santa Cruz), anti-TSG101 (ab83, Abcam), anti-CD63 (ab134045, Abcam), anti-GAPDH (60004-1-lg, Proteintech), secondary antibodies used were anti-Rabbit (7074, CST) and anti-Mouse (7076, CST).

### Reverse transcription and quantitative polymerase chain reaction

The collected RNA of cells and exosomes were extracted using TRIzol reagent (Invitrogen, USA), and complementary DNA (cDNA) was obtained by Transcriptor Reverse Transcriptase (Indianapolis, USA) according to manufacturers’ instructions. qPCR reactions (in 20 μL system) were performed by FastStart Essential DNA Green Master (Roche, Basel, Switzerland). The expression of target gene at RNA levels was normalized to *Gapdh* for comparison and calculated using the 2^−∆∆Ct^. For analysis of siRab4 abundance in exosomes, RNA was isolated and reverse transcribed with miRNA transcriptase kit. U6 served as internal control. All PCR reactions were performed in triplicates. The primer sequences were used in Additional file 1: Table S2.

### Exosome treatment in *Ldlr*^*−/−*^ mice

All animal experiments were approved by the Animal Care and Use Committee of Air Force Medical University. Animal experiments were performed conforming to the Directive 2010/63/EU of the European Parliament. *Ldlr*^*−/−*^ mice (C57BL/6 background) were purchased from the Model Animal Research Center of Nanjing University. All mice were fed with high-fat diet for 8 weeks and then treated with indicated exosomes via tail vein injection at the dose of 4 μg/g body weight once a week for 8 weeks. After 8 weeks of exosomes intervention, all mice were intraperitoneally injected with 1% pentobarbital sodium at 0.1 ml/10 g, and then were killed by cervical dislocation. The main tissues (heart, liver, spleen, lung, kidney and aortic) were separated for subsequent analysis. Blood samples were collected from mice after overnight fasting. All samples were allowed to stand at room temperature for 2 h then centrifuged at 4 °C for 3000*g* for 15 min. The collected supernatants were assayed for AST, ALT, total triglyceride, total cholesterol, LDL cholesterol and HDL cholesterol (Wuhan Servicebio Technology CO, LTD). For histological studies, the main organs (heart, liver, spleen, lung and kidney) were carefully harvested and sectioned for H&E staining. Aorta, aortic roots and liver sections were further stained with Oil-red-O for lipid deposition analysis. The body weights of the mice were recorded weekly for 8 weeks.

### Statistical analysis

Data are expressed as mean ± SEM. One way ANOVA and t test were used for difference comparison by GraphPad prism 9.0. P values < 0.05 were considered statistically significant.

## Supplementary Information

**Additional file 1: Figure S1.** qPCR analysis of knockdown efficiency. **A-L** AML12 cells were transfected with siRNA against genes of interest and relative expression of *Rab33b* (**A**), *Nfs* (**B**), *Rab9* (**C**), *Vps18* (**D**), *Rab22a* (**E**), *Rab35* (**F**), *Rab24* (**G**), *Vps39* (**H**), *Rab14* (**I**), *Rab4* (**J**), *Rab11a* (**K**) and *Tfeb *(**L**). *Gapdh* served as an internal reference gene. Data are expressed as mean $\pm$ SEM of three independent experiments. * *p* < 0.05 by *t*-test. **Figure S2.** qPCR analysis of *Rab31* expression in AML12 cells with *Rab4* knocked-down. AML12 cells were transfected with siRab4 or siNC and expression of *Rab31* was analyzed by qPCR. *Gapdh* as an internal reference gene. Data are expressed as mean $\pm$ SEM of three independent experiments. * *p* < 0.05 by *t*-test. **Figure S3.** Representative flow cytometry analysis of RCMPs uptake by AML12 cells. The AML12 cells were incubated with control or DiI-labeled RCMPs for 6 h, and DiI signal was analyzed by flow cytometry. **Figure S4.** Characterization of Exo^Booster^ from AML12 cells. **A** Representative TEM images of the indicated exosomes from AML12 cells. **B** Size distribution of the indicated exosomes as analyzed by NTA. **C** Western blot analysis of the exosome inclusive and exclusive markers in AML12 cells and derived exosomes. GAPDH served as a loading control. Data shown are representatives from triplicate experiments. **Figure S5.** Fluorescence microscope analysis of Exo^Booster^ biodistribution in vivo**.** Lower magnification images corresponding to Fig. 4E. Scale bar = 100 μm. **Figure S6.** Differential expression of LDLR protein in different tissues from mice. **A–F** Analysis of LDLR protein expression in lung (**A**), kidney (**C**), and heart (**E**) by western blot. Data shown are representative of 3 different experiments. Quantification analysis of western blot results by densitometry in **A**, **C** and **E**, respectively. Data are expressed as mean $\pm$ SEM. ns, no signification. *, *p* < 0.05 by one-way ANOVA. **Figure S7.** *Ldlr*@Exo^Booster^ treatment reduces cholesterol level in *Ldlr*^*−/−*^ mice. **A–D** Plasma total triglyceride (**A**), total cholesterol (**B**), LDL cholesterol (**C**), and HDL cholesterol (**D**) in *Ldlr*^*−/−*^ mice treated as indicated. n = 5. Data are expressed as mean $\pm$ SEM. *p < 0.05 by one-way ANOVA. **Figure S8.** Biocompatibility of indicated exosomes. **A** Body weight change curve in mice with indicated treatments. **B** H&E staining in various tissues. Harvested tissues were sectioned and stained with H&E. No significant histology change was observed in different tissues treated with *Ldlr*@Exo^Ctrl^ or *Ldlr*@Exo^Booster^. Scale bar = 100 μm. **Figure S9.** qPCR analysis of siRab4 sequence in exosomes as indicated. NA, not available as Ct value larger than 38. U6 as an internal control. Data are expressed as mean $\pm$ SEM of three independent experiments. **Table S1.** Sequences of siRNA. **Table S2.** Sequences of PCR primers.

## Data Availability

The data supporting the finding of this study are availability from corresponding author.

## Abbreviations

RCMPs: Red cell membrane particles
Ldlr: Low-density lipoprotein receptor
MVBs: Multivesicular bodies
ESCRT: Endosomal sorting complex required for transport
ILVs: Intraluminal vesicle
NTA: Nanoparticle tracking analysis
EE: Early endosome
LE: Late endosome
RE: Recycling endosome
TGN: Trans-Golgi network
HRS: Hepatocyte growth factor-regulated tyrosine kinase substrate
CLSM: Confocal laser scanning microscope
IVIS: In vivo imaging system
AML12: Alpha mouse liver 12
TBS: Tris buffered saline
