# Supplementary material for: An optimized exosome production strategy for enhanced yield while without sacrificing cargo loading efficiency
Source: J Nanobiotechnology. 2022 Oct 29;20:463. doi: 10.1186/s12951-022-01668-3 (PMC9618217; doi:10.1186/s12951-022-01668-3)
Supplement: Supplementary file 1 — Additional file 1: Figure S1. qPCR analysis of knockdown efficiency. A-L AML12 cells were transfected with siRNA against genes of interest and relative expression of Rab33b (A), Nfs (B), Rab9 (C), Vps18 (D), Rab22a (E), Rab35 (F), Rab24 (G), Vps39 (H), Rab14 (I), Rab4 (J), Rab11a (K) and Tfeb (L). Gapdh served as an internal reference gene. Data are expressed as mean $\pm$± SEM of three independent experiments. * p < 0.05 by t-test. Figure S2. qPCR analysis of Rab31 expression in AML12 cells with Rab4 knocked-down. AML12 cells were transfected with siRab4 or siNC and expression of Rab31 was analyzed by qPCR. Gapdh as an internal reference gene. Data are expressed as mean $\pm$± SEM of three independent experiments. * p < 0.05 by t-test. Figure S3. Representative flow cytometry analysis of RCMPs uptake by AML12 cells. The AML12 cells were incubated with control or DiI-labeled RCMPs for 6 h, and DiI signal was analyzed by flow cytometry. Figure S4. Characterization of ExoBooster from AML12 cells. A Representative TEM images of the indicated exosomes from AML12 cells. B Size distribution of the indicated exosomes as analyzed by NTA. C Western blot analysis of the exosome inclusive and exclusive markers in AML12 cells and derived exosomes. GAPDH served as a loading control. Data shown are representatives from triplicate experiments. Figure S5. Fluorescence microscope analysis of ExoBooster biodistribution in vivo. Lower magnification images corresponding to Fig. 4E. Scale bar = 100 μm. Figur [file 12951_2022_1668_MOESM1_ESM.docx]

**Additional file 1**

**An optimized exosome production strategy for enhanced yield while without sacrificing cargo loading efficiency**

Rongxin Zhang^1,2†^, Te Bu^3†^, Ruidan Cao^4†^, Zhelong Li^3^, Chen Wang^3^, Bing Huang^1, 2^, Mengying Wei^2^, Lijun Yuan^3^, Guodong Yang^2^**^*^**

^1^ College of Life Science, Northwest University, Xi'an 710069, China.

^2^ The State Laboratory of Cancer Biology, Department of Biochemistry and Molecular Biology, Air Force Medical University, Xi’an 710032, China;

^3^ Department of Ultrasound Diagnostics, Tangdu Hospital, Air Force Medical University, Xi’an 710038, China.

^4^ Center of Clinical Aerospace Medicine, Air Force Medical University, Xi’an 710032, China.

^†^ Equal contribution.

***Corresponding author:** Guodong Yang, Department of Biochemistry and Molecular Biology, Air Force Medical University, Changlexi Road NO.169th, 710032, Xi’an, China. Email: yanggd@fmmu.edu.cn; Tel: +862984774516

**Additional Figures and Figure Legends**

**
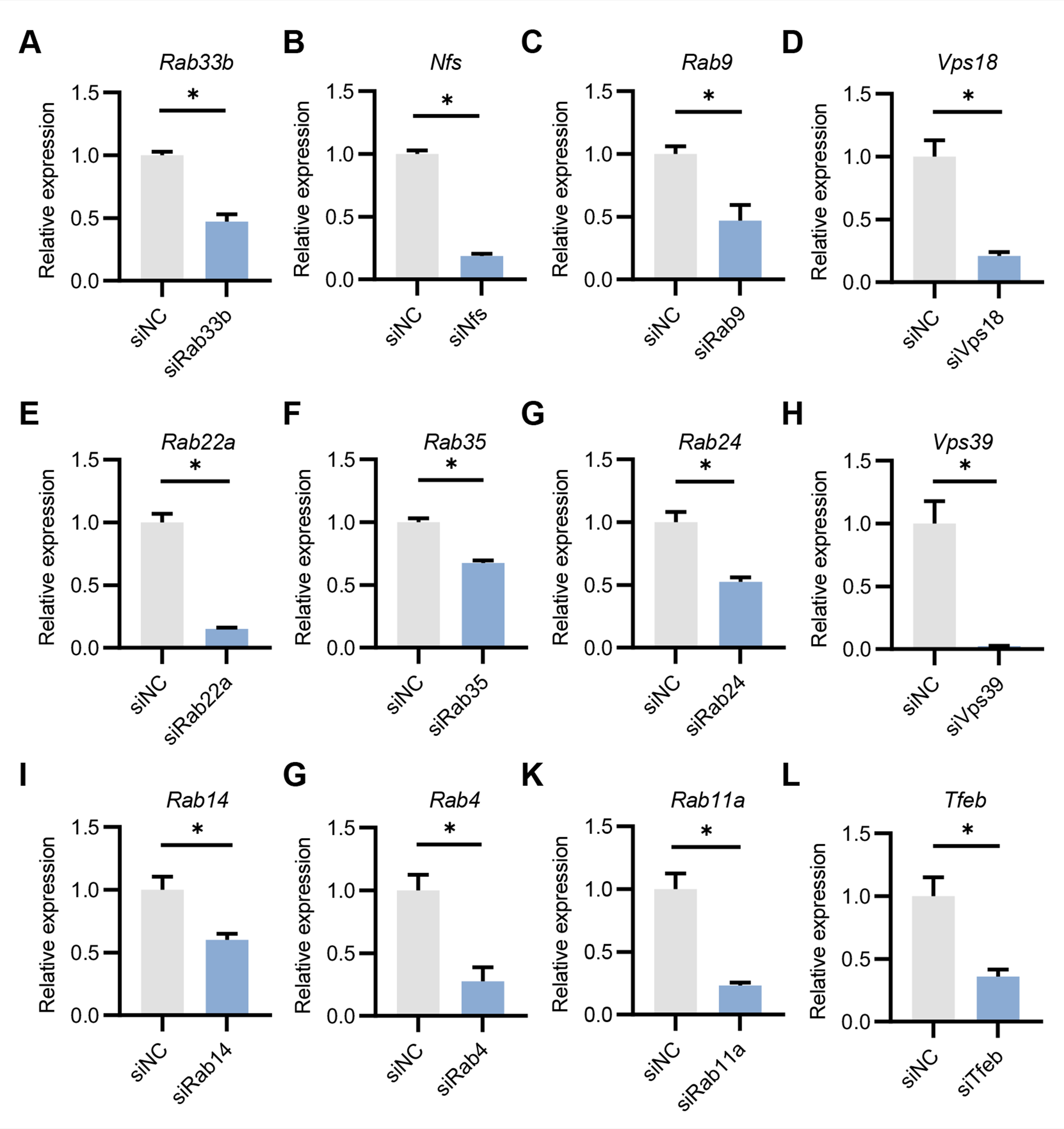
**

**Fig. S1 qPCR analysis of knockdown efficiency. A-L** AML12 cells were transfected with siRNA against genes of interest and relative expression of *Rab33b* (**A)**, *Nfs* (**B)**, *Rab9* (**C)**, *Vps18* (**D)**, *Rab22a* **(E)**, *Rab35* (**F)**, *Rab24* **(G)**, *Vps39* **(H)**, *Rab14* **(I)**, *Rab4* **(J)**, *Rab11a* **(K)** and *Tfeb* **(L)**. *Gapdh* served as an internal reference gene. Data are expressed as mean$\pm$SEM of three independent experiments. * *p*<0.05 by *t*-test.

**
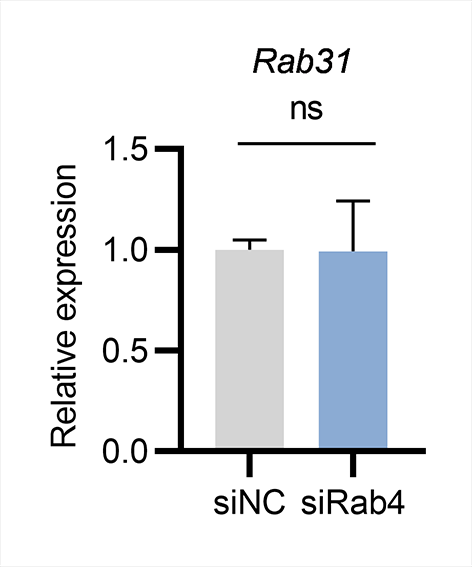
**

**Fig. S2 qPCR analysis of *Rab31* expression in AML12 cells with *Rab4* knocked-down.** AML12 cells were transfected with siRab4 or siNC and expression of *Rab31* was analyzed by qPCR. *Gapdh* as an internal reference gene. Data are expressed as mean$\pm$SEM of three independent experiments. * *p*<0.05 by *t*-test.

**
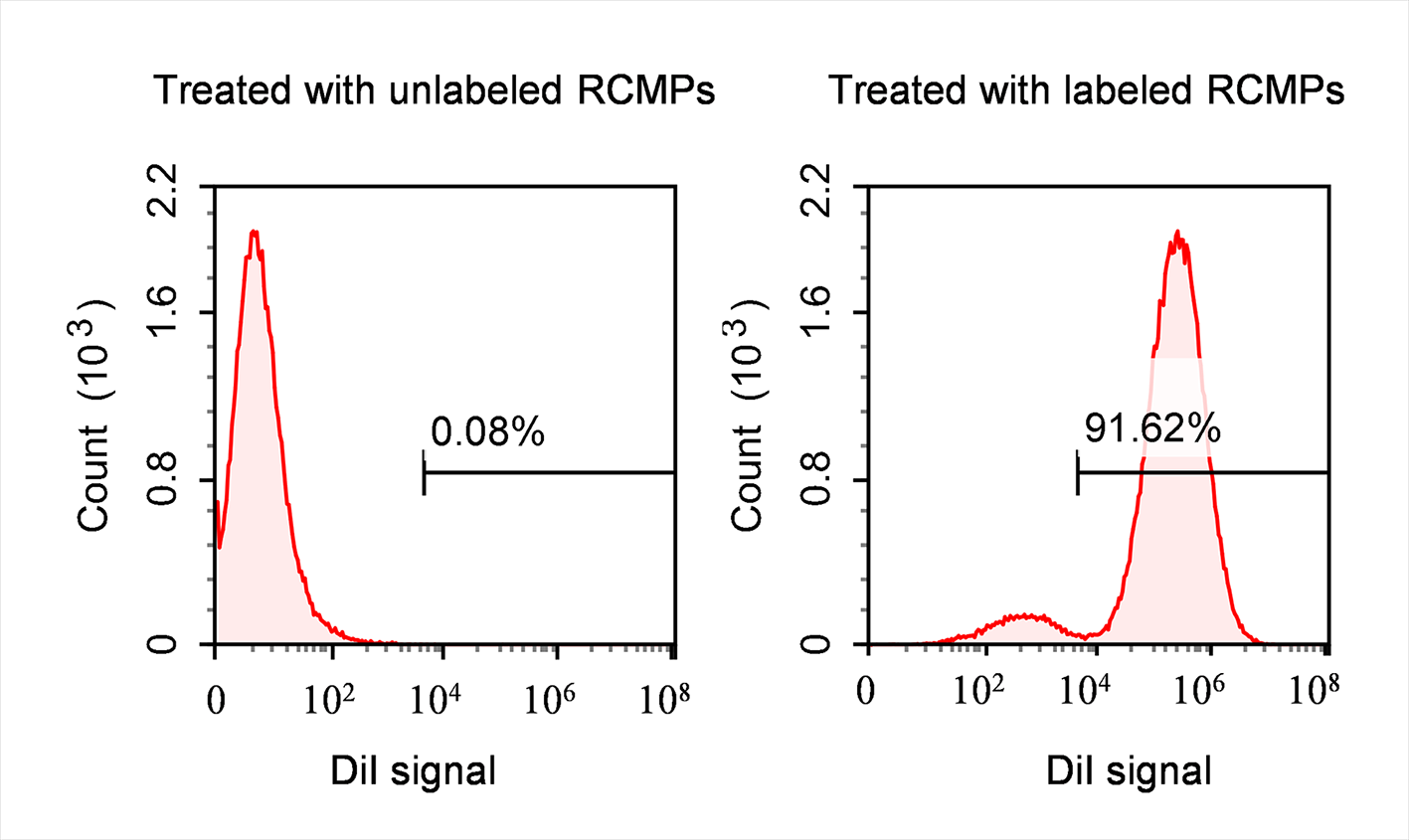
**

**Fig. S3 Representative flow cytometry analysis of RCMPs uptake by AML12 cells.** The AML12 cells were incubated with control or DiI-labeled RCMPs for 6 h, and DiI signal was analyzed by flow cytometry.

**
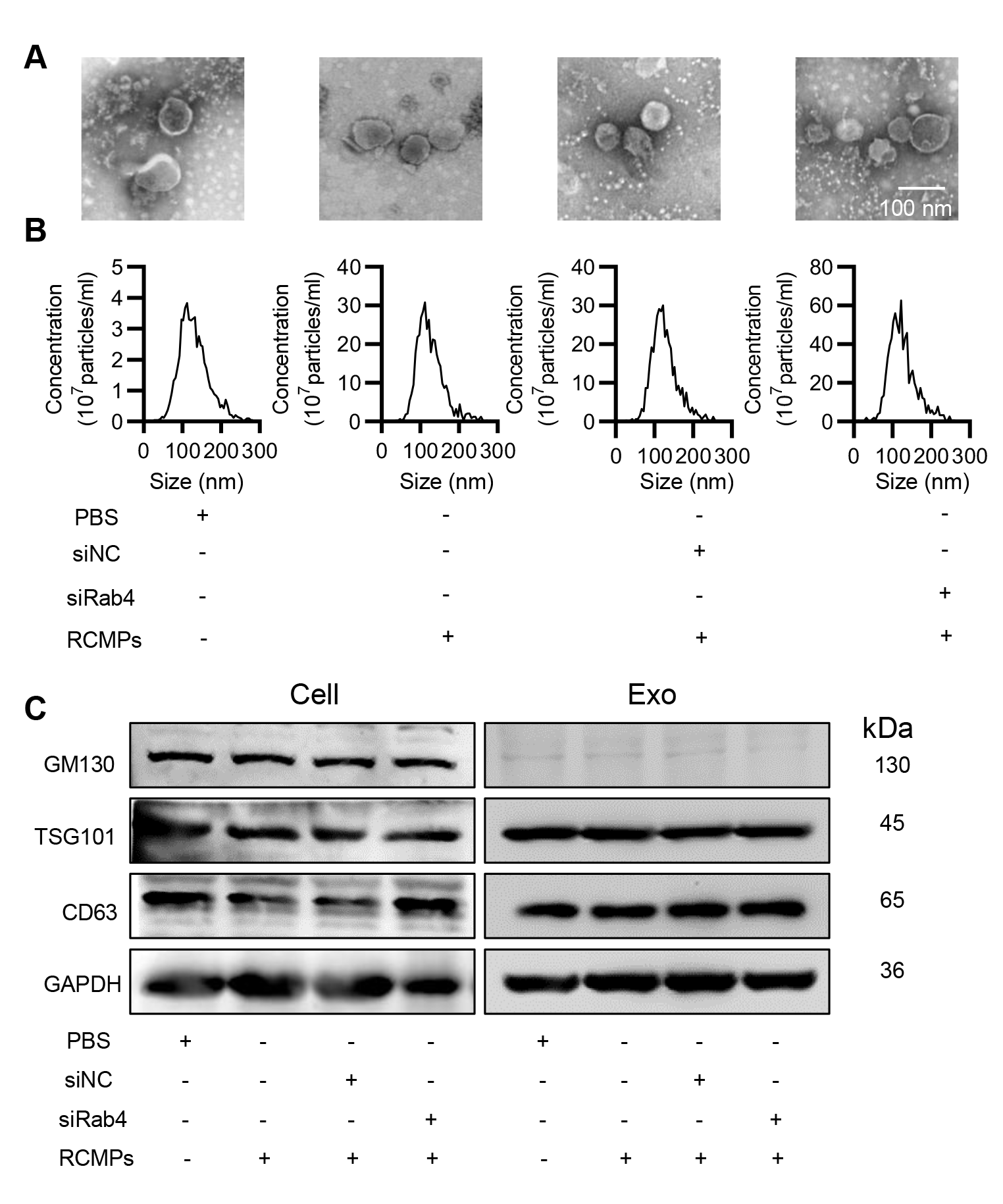
**

**Fig. S4** **Characterization of Exo^Booster^ from AML12 cells.** **A** Representative TEM images of the indicated exosomes from AML12 cells. **B** Size distribution of the indicated exosomes as analyzed by NTA. **C** Western blot analysis of the exosome inclusive and exclusive markers in AML12 cells and derived exosomes. GAPDH served as a loading control. Data shown are representatives from triplicate experiments.

**
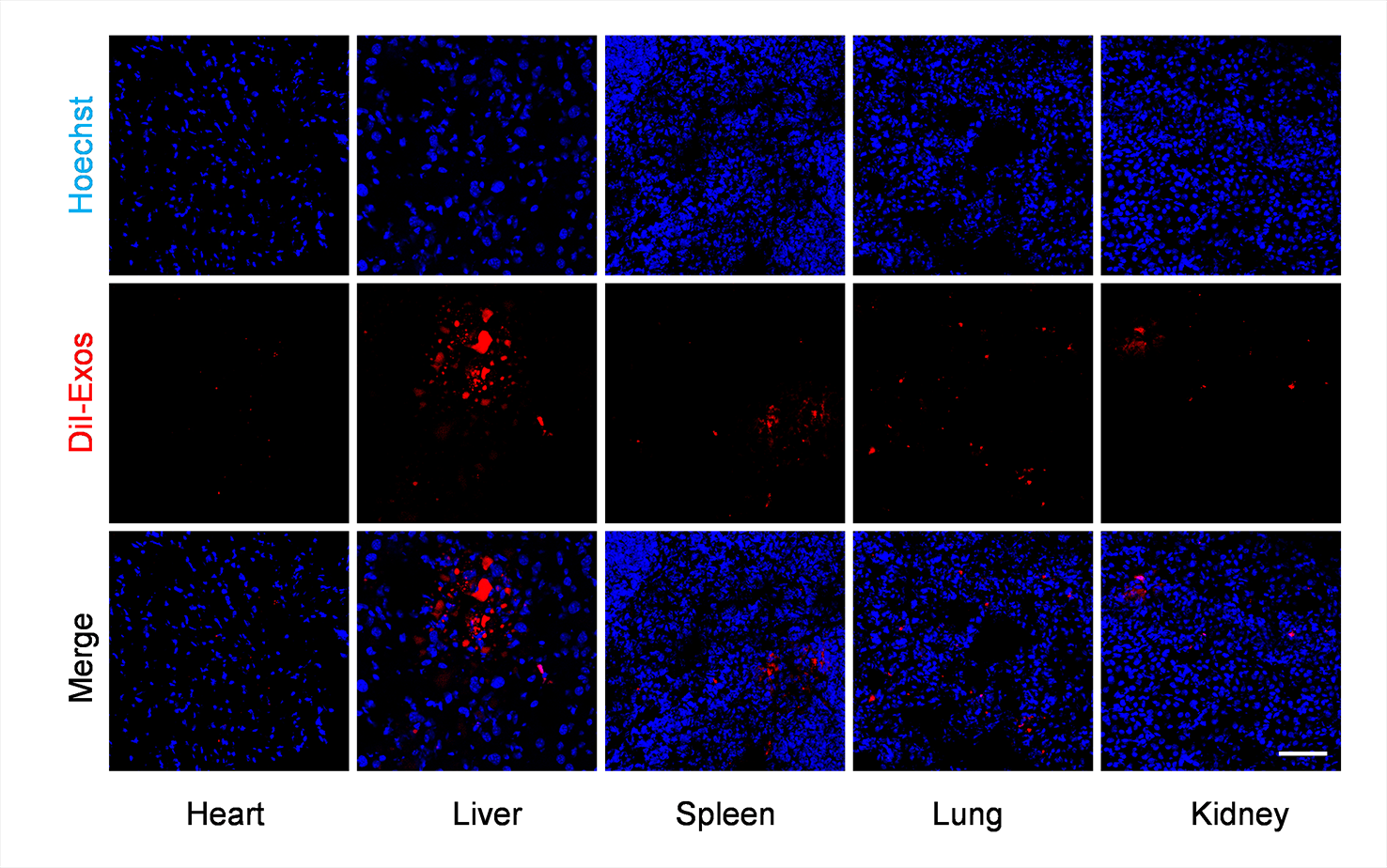
**

**Fig. S5 Fluorescence microscope analysis of Exo^Booster^ biodistribution *in vivo*.** Lower magnification images corresponding to Fig. 4E. Scale bar= 100 μm.


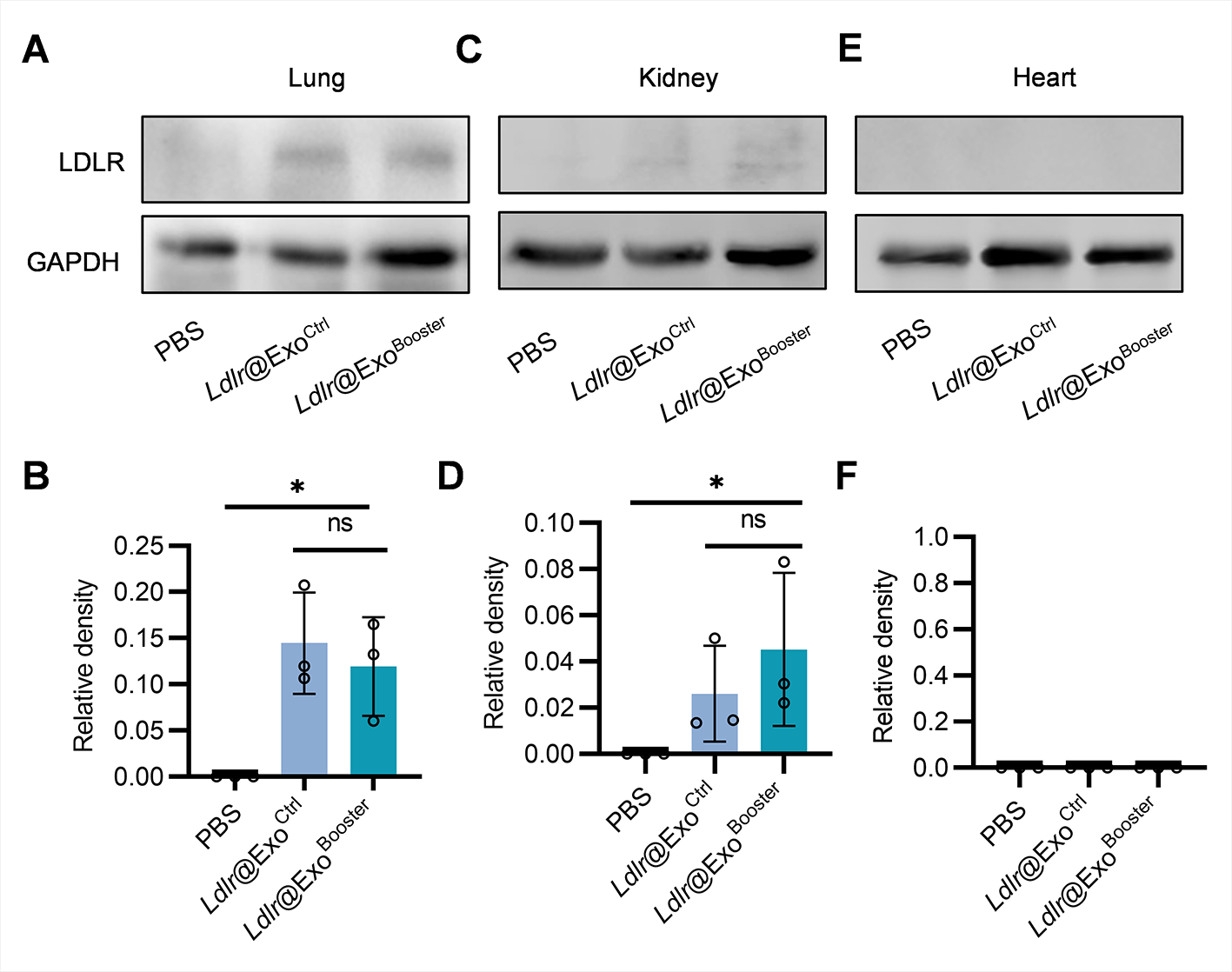


**Fig. S6 Differential expression of LDLR protein in different tissues from mice. A-F** Analysis of LDLR protein expression in lung **(A)**, kidney **(C)**, and heart **(E)** by western blot. Data shown are representative of 3 different experiments. Quantification analysis of western blot results by densitometry in **(A)**, **(C)** and **(E)**, respectively. Data are expressed as mean$\pm$SEM. ns, no signification. *, *p*<0.05 by one-way ANOVA.


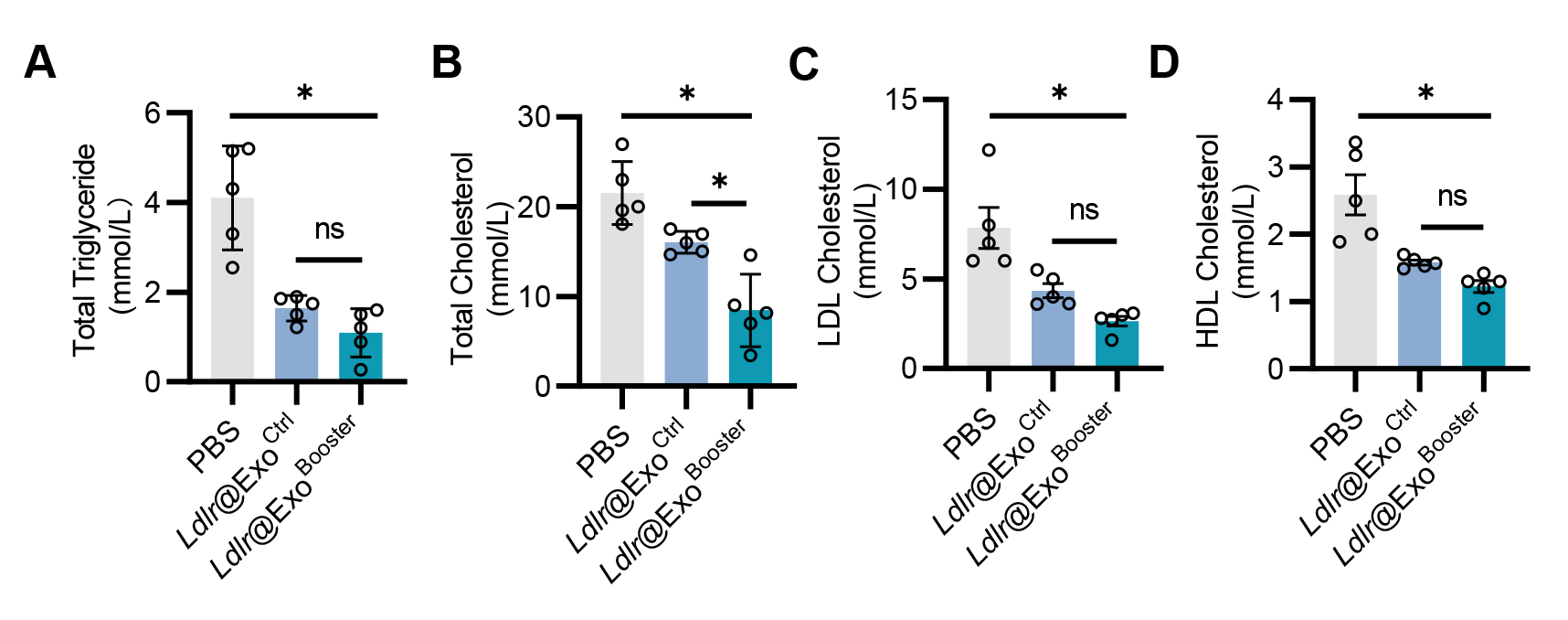


**Fig. S7 *Ldlr*@Exo^Booster^ treatment reduces cholesterol level in *Ldlr^-/-^* mice. A-D** Plasma total triglyceride **(A)**, total cholesterol **(B)**, LDL cholesterol **(C)**, and HDL cholesterol **(D)** in *Ldlr^-/-^* mice treated as indicated. n=5. Data are expressed as mean$\pm$SEM. **p*<0.05 by one-way ANOVA.

**
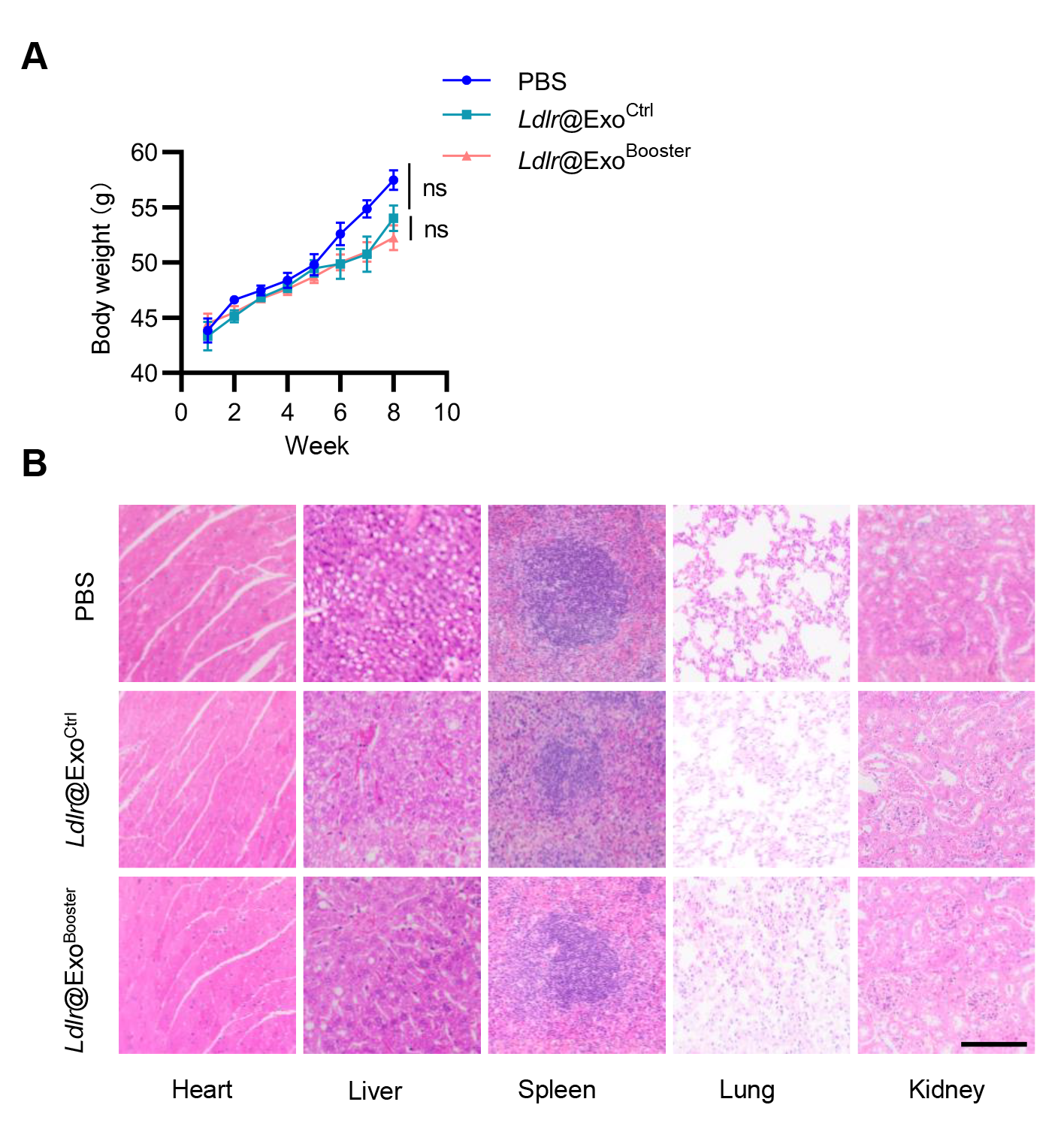
**

**Fig. S8 Biocompatibility of indicated exosomes. A** Body weight change curve in mice with indicated treatments. **B** H&E staining in various tissues. Harvested tissues were sectioned and stained with H&E. No significant histology change was observed in different tissues treated with *Ldlr*@Exo^Ctrl^ or *Ldlr*@Exo^Booster^. Scale bar= 100 μm.


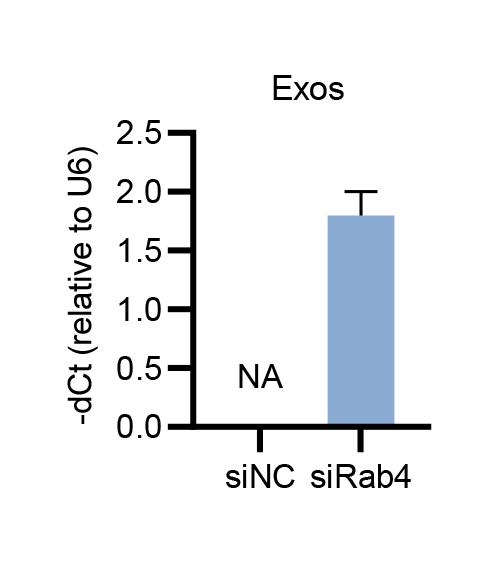


**Fig. S9 qPCR analysis of siRab4 sequence in exosomes as indicated.** NA, not available as Ct value larger than 38. U6 as an internal control. Data are expressed as mean$\pm$SEM of three independent experiments.

**Table S1: Sequences of siRNA**

| Gene name | Sequence (5’-3’) | |
| --- | --- | --- |
|  | sense | antisense |
| NC | UUCUCCGAACGUGUCACGUTT | ACGUGACACGUUCGGAGAATT |
| *Rab4* | GCCAGAACAUCGUCCUUAUTT | AUAAGGACGAUGUUCUGGCTT |
| *Rab33b* | CUGCCUGAUAACAGAAUUATT | UAAUUCUGUUAUCAGGCAGTT |
| *Rab11a* | GGCAGUUCCUACAGAUGAATT | UUCAUCUGUAGGAACUGCCTT |
| *Tfeb* | CAGGCUGUCAUGCAUUAUATT | UAUAAUGCAUGACAGCCUGTT |
| *Rab24* | GCCCAACUCUUUGAAACAUTT | AUGUUUCAAAGAGUUGGGCTT |
| *Rab22a* | CAGCAGCCAUCAUCGUUUATT | UAAACGAUGAUGGCUGCUGTT |
| *Rab14* | GGGAGAGAAUGUAGAAGAUTT | AUCUUCUACAUUCUCUCCCTT |
| *Rab35* | GGGACUACGACCACCUCUUTT | AAGAGGUGGUCGUAGUCCCTT |
| *Rab9* | GCAGGUGUCUACAGAAGAATT | UUCUUCUGUAGACACCUGCTT |
| *Vps18* | CCUGCGUCCAUGUCUAUAATT | UUAUAGACAUGGACGCAGGTT |
| *Vps39* | GGAGCCAGUUGGUAAAGAATT | UUCUUUACCAACUGGCUCCTT |
| *Nsf* | GGGCCAGAAAUCCUUAACATT | UGUUAAGGAUUUCUGGCCCTT |

**Table S2: Sequences of PCR primers**

| Name | Sequences | |
| --- | --- | --- |
|  | Forward (5’-3’） | Reverse (5’-3’) |
| *Rab4* | TTCTTGGTCATCGGAAATGCG | TCTTTGAGCCAAATTCCATTCCT |
| *Rab33b* | AGACGTGCCTGACTTACCG | GTGTCCCACAACTGGATCTTG |
| *Ra*b11*a* | AGGAGCGGTACAGGGCTATAA | ATGTGAGATGCTTAGCAATGTCA |
| *Tfeb* | CCACCCCAGCCATCAACAC | CAGACAGATACTCCCGAACCTT |
| *Rab22a* | GCGCTGAGGGAACTTAAAGTG | ATGCCCCTATGGTTGGATTGA |
| *Rab35* | CCACAATCGGAGTGGATTTCA | CGTCGTAAACCACAATGACCC |
| *Rab9* | ATGGCAGGAAAATCGTCTCTTT | GCATGGTAACAAAATGTCCGTCC |
| *Nsf* | CGGACTATGCAAGCTGCGA | AACCGCACAGTTGCTTAAAGA |
| *Vps39* | CTGCTTCGGATGTACCTGTCA | GGAACTCCGCATGGAGAAGAT |
| *Vps18* | ACGAGGACTCATTGTCCCG | CATACCCAGAATGGGGGATGC |
| *Rab24* | GTGGACGTTAAGGTGGTTATGC | CCCGATGGTGTTCTGATAGGG |
| *Rab14* | ATGGCAACTGCACCGTACAA | CTCCGTGTAACCGCTCTGA |
| *Rab31* | GACACGGGGGTTGGGAAATC | ACAAGGCACGGTTTTGGTCA |
| *Ldlr* | TGACTCAGACGAACAAGGCTG | ATCTAGGCAATCTCGGTCTCC |
| *siRab4* | GCCAGAACATCGTCCTTATTT | Provided in the kit |
| *U6* | CTCGCTTCGGCAGCACA | Provided in the kit |
| *Gapdh* | AGGTCGGTGTGAACGGATTTG | TGTAGACCATGTAGTTGAGGTCA |
